# Tensiomyographic Markers Are Not Sensitive for Monitoring Muscle Fatigue in Elite Youth Athletes: A Pilot Study

**DOI:** 10.3389/fphys.2017.00406

**Published:** 2017-06-16

**Authors:** Thimo Wiewelhove, Christian Raeder, Rauno Alvaro de Paula Simola, Christoph Schneider, Alexander Döweling, Alexander Ferrauti

**Affiliations:** Faculty of Sport Science, Ruhr-UniversityBochum, Germany

**Keywords:** muscle contractile properties, fatigue, training monitoring, junior athletes, high-intensity interval training

## Abstract

**Objective:** Tensiomyography (TMG) is an indirect measure of a muscle's contractile properties and has the potential as a technique for detecting exercise-induced skeletal muscle fatigue. Therefore, the aim of this study was to assess the sensitivity of tensiomyographic markers to identify reduced muscular performance in elite youth athletes.

**Methods:** Fourteen male junior tennis players (age: 14.9 ± 1.2 years) with an international (International Tennis Federation) ranking position participated in this pre-post single group trial. They completed a 4-day high-intensity interval training (HIT) microcycle, which was composed of seven training sessions. TMG markers; countermovement jump (CMJ) performance (criterion measure of fatigue); delayed onset muscle soreness; and perceived recovery and stress were measured 24 h before and after the training program. The TMG measures included maximal radial deformation of the rectus femoris muscle belly (Dm), contraction time between 10 and 90% Dm (Tc) and the rate of deformation until 10% (V10) and 90% Dm (V90), respectively. Diagnostic characteristics were assessed with a receiver-operating curve (ROC) analysis and a contingency table, in which the area under the curve (AUC), Youden's index, sensitivity, specificity, and the diagnostic effectiveness (DE) of TMG measures were reported. A minimum AUC of 0.70 and a lower confidence interval (CI) >0.50 classified “good” diagnostic markers to assess performance changes.

**Results:** Twenty-four hours after the microcycle, CMJ performance was observed to be significantly (*p* < 0.001) reduced (Effect Size [ES] = −0.68), and DOMS (ES = 3.62) as well as perceived stress were significantly (*p* < 0.001) increased. In contrast, Dm (ES = −0.35), Tc (ES = 0.04), V10 (ES = −0.32), and V90 (ES = −0.33) remained unchanged (*p* > 0.05) throughout the study. ROC analysis and the data derived from the contingency table revealed that none of the tensiomyographic markers were effective diagnostic tools for detecting impaired muscular performance in elite youth athletes (AUC, 95% CI, DE%; Dm: 0.46, 0.15–0.77, 35.7%; Tc: 0.29, 0.03–0.59, 35.7%; V10: 0.71, 0.27–1.00, 35.7%; V90: 0.37, 0.10–0.65, 35.7%).

**Conclusion:** The tensiomyographic parameters that were assessed in this study were not sensitive enough to detect muscular performance changes in elite youth athletes.However, due to the preliminary nature of the study, further research is needed to investigate the sensitivity of TMG in this population.

## Introduction

To help elite athletes to progress, modifications in training load are required, particularly adjustments in frequency, duration and intensity (Halson, 2014). This applies equally to both young and adult athletes. As such, many coaches think that in order to achieve success at the senior level, it is necessary to start intensive training well before puberty. This means that many of the youngsters are already training intensively, and for considerable hours, by the time they reach adulthood (Matos and Winsley, 2007).

Compared with adults, youth athletes are able to resist fatigue better and to recover faster during and after exercise (Ratel et al., 2006). Nevertheless, the incidence of overuse symptoms in pediatric and adolescent athletes is increasing (Brenner, 2007). Especially during excessive amounts of high-intensity, repetitive physical activity without adequate rest, the risk of injury, illness, and/or non-functional overreaching is also serious in the youth athlete for several reasons (e.g., the growing bones, ligaments, tendons and cartilage of the young athletes cannot handle as much stress as the mature, passive structures of adults; Matos and Winsley, 2007). Thus, it is important to titrate fatigue appropriately, and to identify youth at risk of overuse (Halson, 2014; Quarrie et al., 2016; Schwellnus et al., 2016; Soligard et al., 2016).

To determine whether a young athlete can tolerate a training program and to minimize the risk of possible negative outcomes, the routine assessment of fatigue and recovery is viewed by many as important (Halson, 2014). In this context, a potentially effective tool for detecting post-exercise muscle fatigue is tensiomyography (TMG), which was introduced as a non-invasive, motivation-independent, and involuntary measure of muscle contractile characteristics (i.e., peripheral fatigue) designed to work without producing additional fatigue (Hunter et al., 2012; Simola et al., 2016b).

The TMG technique is based on the radial deformation of the isolated muscle belly and the time it takes for this action to occur during an isometric twitch contraction evoked by electrical stimulation (Simola et al., 2016b). The key parameters obtained from TMG are muscle displacement, which is representative of muscle tone and contractile force, and the time of the response, which is related to the speed of force generation (Hunter et al., 2012). It is therefore assumed that the effects of exercise-induced damage to muscle structures and the resulting changes in mechanical capacities and performance capabilities (i.e., peripheral fatigue) can be quantified by TMG measures (García-Manso et al., 2011).

A number of studies have been able to demonstrate fatigue-related changes in TMG measures in adults in cases in which exercise-induced muscle damage and/or muscle soreness as well as a decline in performance, peak force and/or rate of force development was observed (Carrasco et al., 2011; García-Manso et al., 2011; Hunter et al., 2012; Simola et al., 2015, 2016a,b; Wiewelhove et al., 2015b). However, we were unable to find any studies that have examined the usefulness of TMG markers to reflect individual changes in post-exercise muscle fatigue in youth athletes. As such, the purpose of the current study was: (1) to investigate changes of TMG variables in elite youth athletes in response to a 4-day high-intensity interval training period, designed to induce a temporary functional overload; and (2) to assess the sensitivity of TMG measures to identify alterations in performance based on individual changes. We hypothesized that the training program leads to an acute increase in perceived fatigue and soreness accompanied by a reduction in physical capacity and that TMG parameters are sensitive to detect muscular performance changes.

## Materials and methods

### Participants

Fourteen elite male junior tennis players (age, 14.9 ± 1.2 years; height: 1.81 ± 0.09 m; body mass, 69.0 ± 11.0 kg; and BMI, 19.01 ± 2.22 kg·m^−1^) with international ITF (International Tennis Federation) ranking positions participated in this study. After being informed about the exercise protocols and all possible risks associated with participation in the investigation, the players and their parents provided written consent to participate in all procedures. Normal electrocardiography findings, as well as the absence of cardiovascular, pulmonary and orthopedic diseases, were confirmed during a preliminary health examination. The study was approved by the ethics committee of the medical faculty of the Ruhr-University Bochum (registration number: 4623-13), and was completed according to the guidelines of the Declaration of Helsinki.

### Experimental design

**Figure 1:**
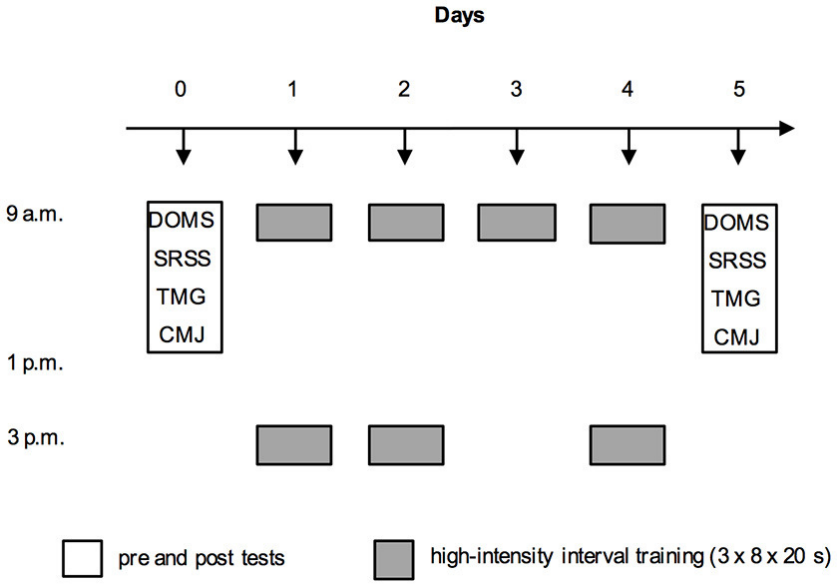
Experimental protocol showing the measurements [delayed onset muscle soreness (DOMS); Short Recovery and Stress Scale (SRSS); tensiomyographic markers (TMG); and countermovement jump performance (CMJ)] and the arrangement of the seven high-intensity interval training sessions during the four-day training period.

A pre-post single group design was used to investigate the sensitivity of TMG markers of muscle fatigue. The youth athletes participated in a 4-day training period, which was composed of seven running-based high-intensity interval training (HIT) sessions. At 72 h prior to the HIT program, all players visited the laboratory for a preliminary health examination, to provide data on anthropometrical characteristics, and to complete the 30-15 Intermittent Fitness Test (30-15_IFT_). TMG markers, countermovement jump (CMJ) performance (criterion measure of fatigue), delayed onset muscle soreness (DOMS), as well as information on perceived recovery and stress, were then measured 24 h prior to the microcycle (pre) as well as 24 h after completing the training program (post) (Figure 1). On both testing days, DOMS, perceived recovery and stress, TMG markers and CMJ were determined (in this order) in the morning between 9 a.m. and 1 p.m. and the testing time was kept constant between days for each player.

### Procedures

#### 30-15 intermittent fitness test

The 30-15 Intermittent Fitness Test (30-15_IFT_) was conducted in a multipurpose indoor training center on a combined elastic flooring system with a PVC surface, and consisted of 30-s shuttle runs interspersed with 15-s passive recovery periods. The speed was set at 8 km·h^−1^ for the first 30-s run and was increased by 0.5 km·h^−1^ at every 45-s stage thereafter. The players were asked to run back and forth between two lines, set 40 m apart, at a pace dictated by an acoustic signal. The test ended when a player was unable to reach a 3 m zone around each line at the moment of the audio signal for three consecutive times. The speed of the last completed stage that was reached by the athlete (V_IFT_) was used to calculate the interval intensity of the HIT protocol, and to estimate athletes VO_2_max according to the following formula: VO_2_max_30−15IFT_ (ml·min·kg^−1^) = 28.3 − 2.15 − 0.741 A − 0.0357 W + 0.0586 A × V_IFT_ + 1.03 V_IFT_, where A stands for age, and W for weight. The mean V_IFT_ and VO_2_max_30−15IFT_ of the athletes were 20.3 ± 0.6 km·h^−1^ and 51.2 ± 1.5 ml·min·kg^−1^ respectively. Previous studies have shown that the 30-15_IFT_ is a valid (V_IFT_ is significantly related to VO_2_max (*r* = 0.68, *p* < 0.05), 10-m sprint time (*r* = 0.63, *p* < 0.05), CMJ height (*r* = 0.65, *p* < 0.05), repeated sprint ability (*r* = 0.88, *p* < 0.001), or the final velocity in the University of Montreal track Test (*r* = 0.79, *p* < 0.001) and reliable (V_IFT_ [km·h^−1^]: intraclass correlation coefficient (ICC) = 0.96) intermittent aerobic fitness test with a typical error (TE) of measurement of approximately 0.3 km·h^−1^ (Buchheit, 2010).

#### Tensiomyography

For the non-invasive assessment of the contractile characteristics of the rectus femoris muscle of the dominant lower limb, TMG was used under laboratory conditions. This technique produces radial displacement of the muscle belly in response to a submaximal (i.e., below voluntary maximal activation) electrical stimulus triggered by a specific electrical stimulator (TMG-S2) that is conducted through the underlying muscle tissue. These displacements are then recorded at the skin surface using a displacement sensor tip with a spring constant of 0.17 N·mm^−1^, together with the TMG-OK 3.0 software (TMG-BMC, Ljubljana, Slovenia).

The sensor was positioned perpendicular to the thickest part of the muscle belly, which was established visually and through palpation during a voluntary contraction, and the self-adhesive electrodes were placed symmetrically approximately 5 cm away from the sensor. Once the exact position for the sensor and electrodes was found, it was marked with a dermatological pen and kept constant during the experimental period (Wiewelhove et al., 2015b). Individual maximal electrical stimulation and Dm were found by progressively increasing the electric current by 20 mA until no further displacement of the muscle belly could be produced. Each stimulation was separated by 10-s intervals to minimize the effects of fatigue and potentiation. The average values from the two maximal twitches was used for further analysis. The rectus femoris muscle was assessed in a supine position, and an internal knee angle of 120° was kept by using supporting pads (Simola et al., 2016b).

The TMG measures included Dm, Tc, V_10_, and V_90_. Dm is equivalent to the maximal radial deformation of the muscle belly which is representative of muscle tone and contractile force. Tc is the deformation time between 10 and 90% Dm. V_10_ and V_90_ can be understood as the rate of deformation development until 10% Dm (10% Dm/Δtime) and 90% Dm (90%Dm/Δtime), respectively. Tc, V_10_, and V_90_ refer to the time and velocities of the muscle radial deformation, which in turn indicate the time and speed of force generation (Simola et al., 2016b). It is assumed that the effects of muscle fatigue, especially on the changes in performance parameters like jump height, can be detected by TMG measures (Hunter et al., 2012; Wiewelhove et al., 2015b): If a decline in jump height is observed then a similar response would be expected by Dm, V_10_, and V_90_, whereas an increase would be shown in Tc. Moreover, Dm, Tc, V_10_, and V_90_were the main parameters in this trial because of sufficient reliability scores (unpublished results, 2013: *n* = 20, Dm [mm]: ICC = 0.87, TE = 0.97, coefficient of variation (CV) = 12.9%; Tc [ms]: ICC = 0.90, TE = 1.86, CV = 5.8%; V_10_ [mm·s^−1^]: ICC = 0.86, TE = 4.00, CV = 12.5%; V_90_ [mm·s^−1^]: ICC = 0.84, TE = 17.89, CV = 13.7%).

#### Jump performance

Following a 5-min standardized warm-up, CMJ were performed on a contact platform (Haynl-Elektronik GmbH, Schönebeck, Germany) with the hands placed on the hips. For CMJ, players dropped down to a self-selected level, before jumping to the maximum height. Flight time was used to calculate jump height. Three CMJs were performed with ~10 s of passive recovery between efforts. The best CMJ value over the three attempts was then computed (Al Haddad et al., 2015). Previously measured reliability scores for the CMJ test were regarded as highly reliable (unpublished results, 2013: *n* = 38, CMJ [cm]: ICC = 0.92, TE = 1.86, CV = 3.7%).

#### Delayed onset muscle soreness

Muscle soreness was assessed using a visual analog scale (VAS). The VAS consisted of a 100 mm line, whose endpoints were labeled as “no pain” (left) and “unbearable pain” (right). Participants were asked to draw a vertical line at a point on the line that best represented their pain at the time of the measurement. Their score was then determined from the distance in mm from the left border of the scale to the point marked (Cleather and Guthrie, 2007).

#### Perceived recovery and stress

Perceived recovery and stress was assessed using the Short Recovery and Stress Scale (SRSS) (Hitzschke et al., 2015). Players were requested to provide responses to eight items on a 0 (i.e., does not apply at all) to 6 (i.e., fully applies) rating scale. Numbers 1–5 on this scale were undefined and were instead used to delineate the degrees of perceived recovery and stress between the two ends of the scale. The items used in this study were “physical performance capability” (PPC) and “muscular stress” (MS). Scores for internal consistencies of the SRSS were previously examined among elite athletes and considered to be sufficient (*n* = 574; α = 0.70–0.76).

### Training program

During the 4-day training period, the athletes completed seven HIT sessions (Table 1). At each session, the players performed three series involving eight intervals, with 20 s of passive recovery between the intervals and 6 min of passive recovery between each series. Each interval was 15 s in duration and consisted of 20-m shuttle runs at 90% V_IFT_. The overall distance, which the athletes had to cover during each interval, differed according to their individual V_IFT_. All sessions were completed in the same indoor training center as the 30-15_IFT_, and were preceded by a standardized continuous 10-min warm-up. To ensure that the intended training intensity was maintained by the players, all sessions were supervised and the individually calculated running distances were controlled.

**Table 1:** Blood lactate concentration before and after training session 1, 3, 5, and 7 as well as athletes' perception of the overall difficulty of each training bout.

**Training day**	**Training session**	**Pre training La**	**Post training La**	**Δ Pre–post training La**	**Session-RPE**
		**(mmol·l^−1^)**	**(mmol·l^−1^)**	***P***	**(0–10)**
1	1 (9 a.m.)	1.35 ± 0.18	11.03 ± 3.13	<0.001	7.5 ± 1.4
	2 (3 p.m.)				7.4 ± 1.2
2	3 (9 a.m.)	1.40 ± 0.23	10.11 ± 3.01	<0.001	7.7 ± 0.7
	4 (3 p.m.)				6.6 ± 1.3
3	5 (9 a.m.)	1.20 ± 0.22	10.45 ± 3.43	<0.001	7.0 ± 1.0
4	6 (9 a.m.)				7.4 ± 1.1
	7 (3 p.m.)	1.46 ± 0.44	11.10 ± 3.50	<0.001	7.2 ± 1.4

*Data are shown as mean ± SD; La: blood lactate concentration; Session-RPE: session rating of perceived exertion*.

Finally, capillary blood samples were obtained from the hyperemized earlobe throughout training sessions 1, 3, 5, and 7 (i.e., before the first interval of the first series and immediately after the last interval of the final series); these were analyzed for blood lactate concentration (La). Blood samples were taken with 20-μl capillaries, hemolyzed in 1-ml micro test tubes, and analyzed by using enzymatic amperometry with the Biosen S-Line Sport (EKF-Diagnostik GmbH, Magdeburg, Germany). In addition, the athlete's perception of the overall difficulty of each training bout as determined 30 min after the completion of an exercise was recorded using a category-ratio scale (Alexiou and Coutts, 2008).

### Statistical analysis

Our statistical analyses were conducted using IMB SPSS Statistics (version 23, IBM Corporation, Amonk, New York, USA) and Microsoft Excel (version 15.17, Microsoft Corp., Redmond, WA, USA). Results are presented as means and standard deviations (SD), and were tested for normal distribution using the Shapiro-Wilk-Test. In cases of non-normal distribution, data was log transformed prior to statistical analysis in order to improve normality and variance homogeneity. All data was first analyzed using the Student's *t*-test for paired data, with significance set at *p* < 0.05. The magnitude of changes between testing days was assessed using the effect size (ES). Threshold values for ES were 0.2 (small), 0.6 (moderate), 1.2 (large), 2.0 (very large), and 4.0 (extremely large) (Hopkins et al., 2009).

Receiver-operating characteristic (ROC) curves were used to investigate the diagnostic accuracy of the TMG measures for the assessment of muscle fatigue in comparison to the criterion measure (i.e., jump performance). A ROC curve plots the true positive rate (i.e., sensitivity) against the true negative rate (i.e., specificity) to produce an area under the curve (AUC). An AUC serves to estimate how high the discriminative power of a test is. The area can have any value between 0.00 and 1.00, and it is a good indicator of the goodness of the test. A perfect diagnostic test has an AUC of 1.00, whereas a non-discriminating test has an AUC of 0.50 (Šimundić, 2008). An AUC > 0.70 and a lower CI > 0.50 have been classified as a “good” benchmark. All ROC curve results were presented as AUC ± 95% CI (Crowcroft et al., 2016).

A 2 × 2 contingency table was used to further evaluate the diagnostic accuracy of TMG measures. The table was composed of horizontal lines to indicate the presence or absence of muscle fatigue (in accordance with changes in TMG parameters) and vertical lines to indicate the “true” condition of a player according to the criterion measure of fatigue. Sensitivity (i.e., the proportion of athletes correctly classified as fatigued), specificity (i.e., the proportion of players correctly categorized as non-fatigued), Youden's index [(sensitivity + specificity) – 1; ranges from 0.00 for a poor diagnostic accuracy and to a value of 1.00 for a perfect diagnostic test] and diagnostic effectiveness (i.e., those who were fatigued and had a positive test plus those who were non-fatigued and had a negative test) were calculated from the constructed table (Šimundić, 2008; Shaikh, 2012).

## Results

La was significantly increased immediately after training sessions 1 [*t*_(13)_ = 11.73, *p* < 0.001], 3 [*t*_(13)_ = 10.83, *p* < 0.001], 5 [*t*_(12)_ = 9.91, *p* < 0.001], and 7 [*t*_(12)_ = 9.96, *p* < 0.001] (Table 1). The players' ratings of the difficulty of each session ranged from 6.6 to 7.7 (i.e., very hard) throughout the study (Table 1).

Twenty-four hours after the training program, CMJ performance was significantly reduced [*t*_(13)_ = −6.45, *p* < 0.001, ES = −0.68] and DOMS, as well as perceived stress (i.e., PPC and MS), had significantly increased [DOMS: *t*_(13)_ = 9.21, *p* < 0.001, ES = 3.62; PPC: *t*_(12)_ = −6.88, *p* < 0.001, ES = −0.66; MS: *t*_(12)_ = 7.76, *p* < 0.001, ES = 1.01] (Table 2). In contrast, Dm, Tc, V_10_, and V_90_ remained unchanged throughout the study [Dm: *t*_(13)_ = −1.42, *p* = 0.178, ES = −0.35; Tc: *t*_(13)_ = 0.13, *p* = 0.896, ES = 0.04; V_10_: *t*_(13)_ = −1.27, *p* = 0.225, ES = −0.32; V_90_: *t*_(13)_ = 1.39, *p* = 0.189, ES = −0.33; Table 2].

**Table 2:** Markers of muscle fatigue before (pre training) and after a 4-day high-intensity interval training program (post training) as well as percentage changes of jump height and muscle contractile properties between testing days.

**Variable**	**Pre training**	**Post training**	**Pre–post training**		
			**%Δ ± Cl**	***P***	***d***
**PERFORMANCE MARKER**					
CMJ (cm)	40.2 ± 4.4	36.9 ± 4.6	−8.1 ± 1.0	<0.001	−0.68
**SUBJECTIVE MARKERS**					
DOMS (mm)	0.4 ± 0.5	3.6 ± 1.9		<0.001	3.62
SRSS PPC	5.4 ± 0.8	3.6 ± 1.2		<0.001	−0.66
SRSS MS	0.5 ± 0.8	3.2 ± 1.5		<0.001	1.01
**MUSCLE CONTRACTILE MARKERS**					
Dm (mm)	8.8 ± 1.9	8.1 ± 2.0	−8.7 ± 0.9	0.178	−0.35
Tc (ms)	31.3 ± 3.9	31.5 ± 4.6	0.2 ± 2.0	0.896	0.04
V_10_ (mm·s^−1^)	33.2 ± 3.9	30.4 ± 7.2	−8.3 ± 3.8	0.225	−0.32
V_90_ (mm·s^−1^)	137.9 ± 34.9	125.5 ± 29.6	−8.6 ± 15.8	0.189	−0.33

*Data are shown as mean ± SD; Cl, 90% confidence limits; d, effect size; CMJ, countermovement jump; DOMS, delayed onset muscle soreness; SRSS, short recovery and stress scale; PPC, physical performance capability; MS, muscular stress; Dm, muscle belly displacement; Tc, contraction time; V_10_, rate of deformation development until 10% Dm; V_90_, rate of deformation development until 90% Dm*.

The determination of the AUC ± 95% CI (Dm: 0.46, 0.15–0.77; Tc: 0.29, 0.03–0.59; V10: 0.71, 0.27–1.00; V90: 0.37, 0.10–0.65) showed that none of the TMG markers had an acceptable discriminative ability (i.e., an AUC > 0.70 and a lower CI > 0.50) in detecting impaired muscular performance in elite youth athletes. The data derived from the contingency table also revealed a poor Youden's index of 0.17 and an insufficient diagnostic effectiveness of 35.7% for all TMG measures. This means that only a third of the individuals were correctly categorized by the tensiomyography method, in relation to muscular performance changes. All sensitivity data are reported in Table 3.

**Table 3:** Accuracy of tensiomyographic markers of muscle fatigue in relation to the criterion measure.

**Variable**	**AUC (95% Cl)**	**Sensitivity (%)**	**Specificity (%)**	**Youden's index**	**DE (%)**
Dm (mm)	0.46 (0.15 – 0.77)	33.3	50.0	0.17	35.7
Tc (ms)	0.29 (0.03 – 0.55)	33.3	50.0	0.17	35.7
V_10_ (mm·s^−1^)	0.71 (0.27 – 1.00)	33.3	50.0	0.17	35.7
V_90_ (mm·s^−1^)	0.37 (0.10 – 0.65)	33.3	50.0	0.17	35.7

*Cl, confidence limits; AUC, area under curve; DE, diagnostic effectiveness; Dm, muscle belly displacement; Tc, contraction time; V_10_, rate of deformation development until 10% Dm; V_90_, rate of deformation development until 90% Dm*.

## Discussion

To the authors' knowledge, this is the first trial that examined the sensitivity of tensiomyographic markers of muscle fatigue in elite youth athletes. The major finding of this investigation was that the TMG parameters used were not sensitive enough to detect significant muscular performance changes and, consequently, muscle fatigue induced by a 4-day HIT shock microcycle. As such, these results suggest that tools other than TMG should be implemented in both the scientific and applied environments as a more effective means to measure muscle fatigue in youth athletes. Furthermore, collected data showed that the training program caused an acute increase in symptoms of fatigue. These results confirm the findings of other research studies that have demonstrated significant changes in measures of fatigue following HIT cycles (Halson et al., 2002; Wiewelhove et al., 2015b). Finally, the present findings show that the HIT protocol was extremely demanding, since the players produced high La levels of up to 11.10 mmol·L^−1^, although young athletes usually accumulate less blood lactate than adults do during intensive exercise (Armstrong and McManus, 2011).

In this study, countermovement jump performance (ES = −0.68; percentage change = −8.1%) and perceived physical performance capability (ES = −0.66) moderately decreased after the training period, while delayed onset muscle soreness (ES = 3.62) and muscular stress (ES = 1.01) were very largely and moderately increased. Since the CV of the CMJ performance was 3.7%, the magnitude of the change in muscular performance can be considered to be of practical relevance. These results are in line with those of other research studies that have demonstrated a significant increase in the symptoms of fatigue among adult athletes following intermittent high-intensity exercise and partially confirm our hypothesis (Thompson et al., 1999; Halson et al., 2002; Wiewelhove et al., 2015a,b).

Historically, potential mechanisms related to the plethora of fatigue symptoms following HIT can be classified into two categories: (1) central factors involving the central nervous system and nervous pathways; and (2) peripheral factors occurring within the muscle itself. However, the basic assumption is that subsequent fatigue after high-intensity dynamic exercise would account for about 80% from a peripheral origin (Ratel et al., 2006). This includes physical signs such as disrupted sarcomeres and/or damage to components of the excitation-contraction coupling system. During these events, the transient symptoms include prolonged reductions in maximal force, ground reaction force, stretch-reflex sensitivity, muscle joint stiffness regulation and, thus, a decrease in muscular performance as well as an increase in muscle soreness and perceived fatigue (Eston et al., 2003). These symptoms would be consistent with our findings regarding CMJ performance, DOMS, and perceived recovery and stress following the HIT training program.

In contrast, however, we observed no significant alterations in TMG parameters following completion of the HIIT program. Moreover, none of the TMG markers evaluated showed sufficient sensitivity to the detection of the altered muscular performance induced by the HIT microcycle. This is in contradiction with our hypothesis, as well as with a variety of recent studies. For example, Simola et al. (2015) were able to demonstrate that the decreases in maximal voluntary contraction in a half-squat isometric exercise after different dynamic squat training protocols were accompanied by reductions in Dm, V_10_, and V_90_. Additionally, following a 6-day running-based HIT-microcycle, Wiewelhove et al. (2015b) observed similar things: namely, that jump and sprint performance had significantly declined together with a simultaneous increase in creatine kinase, muscle soreness, and Tc. Carrasco et al. (2011), García-Manso et al. (2012), Hunter et al. (2012), and MacGregor et al. (2015), as well as Raeder et al. (2016), were also able to show that TMG parameters significantly followed other standard exercise-induced muscle damage and fatigue responses (i.e., changes in maximal voluntary contraction, physical performance, passive muscle tension, creatine kinase, and/or muscle soreness).

The TMG technique involves neuromuscular electrical stimulation, which is delivered using electrodes placed on the skin over a muscle belly. The subsequent muscle response is therefore predominantly generated through the activation of motor axons beneath the stimulating electrodes (Bergquist et al., 2012). Mechanisms of post-exercise muscle fatigue responsible for changes in TMG parameters are thus located peripherally, rather than centrally. In this respect, Hunter et al. (2012) and MacGregor et al. (2015) suspected that a medium-term fatigue related decrease in Dm as well, as an increase in time and velocities of the muscle radial deformation, can be mainly explained by events that occur during primary and secondary exercise-induced muscle damage. This may include excitation-contraction coupling impairment, the redistribution of sarcomere lengths, the loss of membrane integrity and the destruction of cellular structures, which in turn result in an increase in tone or stiffness of the muscle, swelling of the limb, and/or a reduction in the muscle's ability to generate force. Accordingly, the decline in CMJ performance as well as changes in TMG parameters are probably linked to the same occurrences. Nevertheless, TMG markers were not able to effectively track the reduced muscular performance and instead remained unchanged during the training period. The precise reasons for this disparity are unclear; however, there are a number of possible explanations.

As already mentioned above, it is assumed that the immediate and prolonged loss of physical performance capacity (e.g., jump performance) after HIT is primarily caused by factors at or distal to the neuromuscular junction (i.e., peripheral fatigue). Ratel et al. (2015), however, showed that youth athletes experienced no apparent peripheral fatigue and had higher central fatigue than adults similarly induced by repeated maximal contractions. They hypothesized that the greater central fatigue that occurred in the children and adolescents could be related to a strategy of the central nervous system aimed at limiting the recruitment of motor units to prevent any extensive peripheral fatigue. This hypothesis is supported by Chen et al. (2014) as well as Marginson et al. (2005), who observed that adolescent boys have a significantly smaller extent of exercise-induced muscle damage compared with that of adults. They also explained the milder symptoms of exercise-induced muscle damage in youth athletes was due to a greater reliance on slow-twitch muscle fibers and a greater flexibility. In addition, the authors assumed that the ability of adolescents to produce more relative strength than the men at long muscle lengths possibly led to less of an overextension of sarcomeres during damaging exercise bouts. Overall, this may be why a decrease in CMJ performance but no alterations in TMG parameters, which are primarily influenced by peripheral fatigue, could be observed.

Besides, as with any type of physiological measurement, there is a degree of uncertainty or noise in the tensiomyographic test results. In this regard, the findings of a preliminary investigation of our own working group exhibited an excellent level of relative reliability for Dm, Tc, V_10_, and V_90_ (ICC = 0.84–0.90). Ditroilo et al. (2013) assessed the long-term stability of TMG across a variety of muscle conditions and came to similar conclusions. However, assessing the sensitivity of TMG measures to identify alterations in performance is based on the interpretation of the individual percentage changes in Dm, Tc, V_10_, and V_90_ in relation to their absolute reliability (i.e., CV), and not to their relative reliability. This means that to make sure that the given magnitude of observed changes is real and physiologically significant, it is necessary to take into account the level of absolute reliability of the TMG parameters. In this context, Ditroilo et al. (2011) and Ditroilo et al. (2013), as well as the results of our preliminary investigation (CV = 5.8–13.7%), have shown that the level of absolute reliability among the TMG parameters examined was questionable. Therefore, since noise of the tensiomyographic test results is quite high, the assessment of individual changes in TMG markers is problematic. This may be why TMG measures were found to be insufficiently sensitive to adequately detect muscle fatigue in youth athletes.

Finally, some limitations of the study design must be considered. First, there was no control group and the sample size was small, which, in particular, weakens the meaningfulness of the ROC analysis and the data derived from the contingency table. However, we have provided reliability data to indicate typical variation in all measures and effect sizes to indicate trends in the data. In addition, recruiting high-level athletes for standardized research approaches is problematic, especially due to the reluctance of such populations to deviate from their normal training routine. It was therefore not feasible to carry out a controlled trial with an appropriate sample size. Previous studies have also shown that there is acute fatigue following a HIT cycle (Halson et al., 2002; Wiewelhove et al., 2015b), so we believe that the decrements in physical capacity following the treatment were not coincidental. Regardless of this, the purpose of the study was to assess if TMG markers were sensitive to performance decrements, so a control group was not imperative.

Secondly, only the contractile characteristics of the rectus femoris muscle were measured through TMG; however, the effect of muscle fatigue on other muscles that are involved in running-based activities (e.g., gluteus maximus, biceps femoris long head, vastus medialis, vastus lateralis, soleus, or tibialis anterior) might be different. Consequently, it remains to be seen whether TMG markers of other muscles than the recuts femoris have the sensitivity needed to detect reduced muscular performance in elite youth athletes. Nevertheless, it is still important to note that TMG is intended to be a field based measure (Ditroilo et al., 2013). Therefore, the number of different muscles that are examined for the detection of muscle fatigue should be kept as low as possible; otherwise, TMG measurements would take considerably longer, making it impractical for its proposed purpose.

Thirdly, one could question why the recuts femoris muscle was selected for analysis in the present study. Sloniger et al. (1997), for example, showed that the muscles or muscle groups most activated during horizontal running were the adductors, semitendinosus, gracilis, biceps femoris and semimembranosus. However, other studies have also shown that the rectus femoris, together with the vastus lateralis, vastus intermedius, and vastus medialis, are the largest contributors to braking the body mass center during running and to absorbing the shock of the impact during each stance phase (Montgomery et al., 1994; Hamner et al., 2010). This is to be considered, since the training program consisted of 20-m shuttle runs in which the athletes had to undergo numerous changes in directions (COD). Each COD results in considerable decelerations and consequently, in eccentric contractions of the recuts femoris muscle needed in order to brake the body mass center. Therefore, it can be assumed that the recuts femoris was used intensively during the four-day HIT shock microcycle.

Fourthly, the previously reported reliability scores of the tensiomyographic markers used in this study were determined in a study that involved grown-up athletes. However, due to the smaller muscle size of adolescents, the precise positioning of the TMG sensor and the electrodes is more problematic than in adults, and the likelihood that the TMG measures are affected by involuntary contractions of other, nearby muscles is increased. This could further reduce the reliability of TMG when used in young athletes and, as a result, their sensitivity for monitoring muscle fatigue.

## Conclusion

The number of youth athletes participating in organized athletic activities is increasing. Additionally, the combination of increased exposure and decreased preparedness for sports participation has led to an epidemic of sports-related overuse and injuries in this population (Carter and Micheli, 2011). In this context, TMG was introduced as a potentially effective tool to determine whether athletes can tolerate a certain amount of physical activity and to minimize the risk of possible negative outcomes.

To the best of our knowledge, this is the first study that examined the sensitivity of tensiomyographic markers of muscle fatigue as a means to identify individual alterations in performance in elite youth athletes. The major finding of this study was that the TMG parameters considered were not sensitive enough to detect significant muscular performance changes and, consequently, any muscle fatigue induced by intensified training. Although the precise reasons for these findings are unclear, the performance decrements observed in the youth athletes may be from central rather than peripheral fatigue, which is likely to be undetectable with TMG. Furthermore, TMG measurements are noisy, which makes it difficult to determine if individual changes in tensiomyographic markers are meaningful. These two factors may limit the sensitivity of tensiomyographic markers for monitoring muscle fatigue in youth athletes and highlight that other tools should be implemented to monitor and titrate fatigue appropriately in this population and to identify individuals at risk of overuse injury. However, due to the preliminary nature of the study, which involved a single group design and low number of subjects, further research is needed to investigate the sensitivity of TMG in youth athletes.

## Author contributions

TW and AF, Conceived and designed the experimental design; TW, CR, RD, CS, AD, Performed the experiments; TW and CS, Analyzed the data; TW, CR, RD, CS, AD, and AF, Contributed reagents, materials and/or analysis tools; TW and AF, Wrote the paper.

### Conflict of interest statement

The authors declare that the research was conducted in the absence of any commercial or financial relationships that could be construed as a potential conflict of interest.
